# Functional Analysis of the Pathogenicity-Related Gene *VdPR1* in the Vascular Wilt Fungus *Verticillium dahliae*

**DOI:** 10.1371/journal.pone.0166000

**Published:** 2016-11-15

**Authors:** Ya-Lin Zhang, Zhi-Fang Li, Zi-Li Feng, Hong-Jie Feng, Yong-Qiang Shi, Li-Hong Zhao, Xi-Ling Zhang, He-Qin Zhu

**Affiliations:** State Key Laboratory of Cotton Biology, Institute of Cotton Research of Chinese Academy of Agricultural Sciences, Anyang, Henan, 455000, China; National Key Laboratory of Crop Genetic Improvement, CHINA

## Abstract

*Verticillium dahliae* Kleb., the causal agent of vascular wilt, can seriously diminish the yield and quality of many crops, including cotton. The pathogenic mechanism to cotton is complicated and unclear now. To screen pathogencity related genes and identify their function is the reliable way to explain the mechanism. In this study, we obtained a low-pathogenicity mutant vdpr1 from a T-DNA insertional library of the highly virulent isolate of *V*. *dahliae* Vd080, isolated from cotton. The tagged gene was named pathogenicity-related gene (*VdPR1*). The deletion mutant ΔVdPR1 did not form microsclerotia and showed a drastic reduction in spore yield and mycelial growth, compared to wild type. Also, ΔVdPR1 showed significantly lower protease and cellulase activities than those of wild type. Complementation of the mutant strain with *VdPR1* (strain ΔVdPR1-C) almost completely rescued the attributes described above to wild-type levels. The knockout mutant ΔVdPR1 showed delayed infection, caused mild disease symptoms, formed a smaller biomass in roots of the host, and showed compromised systemic invasive growth in the xylem. These results suggest that *VdPR1* is a multifaceted gene involved in regulating the growth development, early infection and pathogenicity of *V*. *dahliae*.

## Introduction

Cotton (*Gossypium hirsutum* L.) is the most important source of natural textile fibers worldwide, and is also a significant oilseed crop. Verticillium wilt severely damages cotton production, and strategies to minimize this disease are a high priority. To date, Verticillium wilt is managed in cotton by the use of resistant or tolerant varieties. *Verticillium dahliae* is a soil-borne phytopathogenic fungus that causes vascular wilt diseases in a wide variety of crop plants, resulting in extensive economic losses [1–3]. This pathogen is difficult to control because of its wide host range and its long-living dormant microsclerotia [4, 5].

Annotated whole genome sequences for the two most prevalent *Verticillium* pathogens, *Verticillium dahliae* VdLs.17 and *Verticillium albo-atrum* VaMs.102, have been published online [2]. Since then, there have been several studies on the pathogenicity of *V*. *dahliae*, and several genes essential for its pathogenicity have been identified. Microsclerotia, the dormancy structure of *V*. *dahliae*, are important for the life cycle of this pathogen. Although microsclerotia development has been studied at the morphological level, little is known about the molecular pathways involved in this process [6]. Genes associated with the formation of microsclerotia include *VMK1*, *VDH1*, *Vdgrp1*, *VdSNF1*, *VdNLP*, and *VdPR3*. All of these genes have been shown to positively regulate microsclerotia development, and knock-outs of each of these genes resulted in reduced microsclerotia formation [3, 6–11]. *VdPKAC1* and *VGB* have been shown to negatively control microsclerotia development, and disruption of each of them improved microsclerotia development [12, 13].

Verticillium wilt disease is caused by the systemic colonization of plant vascular tissues by *V*. *dahliae*, and pathogenicity-related genes directly or indirectly participate in this process [14]. Successful penetration of the plant cells is the first step in colonization and the induction of pathogenicity in *V*. *dahliae*. *Vta2*, which is transcriptional activator controlling the expression of genes encoding adhesins and secreted proteins, is a major regulator of fungal pathogenesis, and controls infection of the host-plant root at the initial phase [15]. In addition to *Vta2*, *VdMsb*, which encodes a transmembrane mucin, has also been implicated in the invasive growth and adhesive capacity of *V*. *dahliae* [16]. In plants, the cell wall is a natural barrier that plays an important role in defense against pathogens. Pathogenic fungi must overcome the cell wall barrier to infect the plant; therefore, the activities of hydrolytic cell wall-degrading enzymes (CWDE) are related to pathogenicity. The sucrose non-fermenting protein kinase (encoded by *VdSNF1*) controls the activities of pectinases and galactases, and was shown to be required for virulence. The growth of a strain with a mutated *VdSNF1* was significantly reduced when grown with pectin and galactose as the carbon sources [9]. The specific secreted protein (encoded by *VdSSP1*) showed a similar function to that of VdSNF1 to utilize carbon sources such as pectin and starch, and was shown to be essential for the penetration of cotton plant roots [17]. After successfully invading plant-host cells, the fungal mycelium advances intercellulary through the root cortex into the xylem vessels. During this process, the fungus has only a limited supply of nutrients and amino acids [18]. *VdThi4* is required for growth under vitamin B1-limiting conditions, and a mutant lacking this gene showed impaired growth on thiamine-free medium. The ΔVdTHI4 deletion strain was still able to invade plants through the roots, but did not produce disease symptoms; therefore, *VdThi4* is required for the pathogenicity of *V*. *dahliae* [18]. Following the adhesion and penetration of *V*. *dahliae*, *VGB*, *VdSge1* and other genes directly/indirectly related to pathogenesis are induced. The pathogen moves to neighboring plant cells and infects other tissues, ultimately causing the death of the host plant [13, 19]. *VGB* encodes a G protein β-subunit, and disruption of VGB in *V*. *dahliae* resulted in mutants with severely impaired virulence to tomato and eggplant, but increased microsclerotia formation and conidiation. The mutants also produced less ethylene than did the wild-type strain. Analyses of these mutants indicated that pathogenicity was linked to cAMP-PKA signaling pathways [13]. Besides *VGB*, *VdSge1* is a transcriptional regulator, differentially regulates expression of effector genes, *VdSge1* deleted strains showed reduced radial growth, lower conidia production, and lost pathogenicity to tomato [19].

In our previous study, we constructed an *Agrobacterium tumefaciens*-mediated transformation (ATMT) insertional mutant library of the highly virulent *V*. *dahliae* strain Vd080 [20]. After two rounds of selection, we obtained 25 mutants with significantly reduced pathogenicity and a single-copy insertion [21]. One of them, vdpr1, showed significantly lower virulence to cotton and produced no microsclerotia. In this study, targeted deletion of *VdPR1* from the *V*. *dahliae* wild-type isolate Vd080 revealed its roles in fungal growth and conidiation, microsclerotia development, extracellular enzyme activity, and virulence.

## Materials and Methods

### Fungal strains and culture conditions

The *V*. *dahliae* strain Vd080, which was isolated from cotton collected in Xinji, Hebei, China (37°56′N, 115°15′E), was used in this study. The trail plots were public research sites under the management of Chinese Academy of Agriculture Sciences, without infringing private property. This isolate and its transformants were single-spore isolated and stored at −80°C in 20% (v/v) glycerol. The low-pathogenicity mutant vdpr1 was selected from the T-DNA insertional library of the *V*. *dahliae* strain Vd080. Fungal strains were cultured on potato dextrose agar (PDA) medium to observe biological characteristics. To induce conidia formation for the infection assays, isolates were incubated in liquid Czapek-Dox medium (30 g/L Sucrose, 2 g/L NaNO_3_, 0.5 g/L MgSO_4_-7H_2_O, 0.5 g/L KCl, 0.02 g/L FeSO_4_-7H_2_O, and 1 g/L K_2_HPO_4_) at 25°C with shaking at 150 rpm [22]. *Escherichia coli* trans1-α and *Agrobacterium tumefaciens* strain AGL-1 were used in transformation procedures [23].

### *VdPR1* gene cloning and bioinformatics analysis

Genomic DNA was isolated using the cetyl-trimethylammonium bromide (CTAB) method [24]. A thermal asymmetric interlaced PCR (TAIL-PCR) was used to clone the genomic DNA flanking the T-DNA insert in the insertion mutant vdpr1 [25]. The right border primers (R-SP_1_, R-SP_2_, and R-SP_3_) and left border primers (L-SP_1_, L-SP_2_, and L-SP_3_) were designed in our previous study [21]. We used four arbitrary degenerate primers as described previously [26]. TAIL-PCR amplifications were carried out using a genome walking kit (Takara, Dalian, China) and sequenced by the Genewiz Corporation (Nanjing, China). The innermost specific primer, either R-SP_3_ or L-SP_3_, was used as the sequencing primer. The T-DNA insertion site was identified by comparing sequences around the T-DNA with those in the VdLs.17 genome database. Based on the information obtained, the full-length *VdPR1* sequence containing the entire ORF was cloned from strain Vd080 genomic DNA and cDNA using ORF-specific primers (Table 1).

**Table 1:** Primers used in this study.

Primer name	Primer sequence (5′-3′)
Cloing of *VdPR1*	
ORF-F	ATGAAGTTTTCCCCCTCA
ORF-R	TCATCCATCCTGCCCGAA
Construction of *VdPR1* knockout vector	
HPH-F	TTGAAGGAGCATTTTTGGGC
HPH-R	TTATCTTTGCGAACCCAGGG
P1	ATCTCGTGCCGCTTGGTACA
P3	GCCCAAAAATGCTCCTTCAAGTAGTGTGCGTGAACGTCGC
P4	CCCTGGGTTCGCAAAGATAAAGCGGTCTTGAGTATGCGCT
P6	TCCAACGAGGTAGGCAAACGA
P2	GGGGACAAGTTTGTACAAAAAAGCAGGCTTTGCAATGCCGTGCCAAAAC
P5	GGGGACCACTTTGTACAAGAAAGCTGGGTGGACCACGTGTGGAAGACCT
Test-F	TACAGCCAAGTCAACCGAGC
Test-R	CGTAGAGGAAGGCGCGATGA
Construction of *VdPR1* complementary vector	
COM-F	CCCGGGTCGTCAAGGATTGCTCGCCA
COM-R	TGTACAGGACCACGTGTGGAAGACCT
GFP-F	ATGCCGTGAGTGATCCCGGCGGC
GFP-R	ATGGTGAGCAAGGGCGAGGAGCT
*VdPR1* transcript detection	
RT-F	TACAGCCAAGTCAACCGAGC
RT-R	CGTAGAGGAAGGCGCGATGA
Vdβt-F	AACAACAGTCCGATGGATAATTC
Vdβt-R	GTACCGGGCTCGAGATCG
qPCR quantification of fungal biomass	
Act-F	CCTATGTTGCCCTGGACTATGAGC
Act-R	GGACAACGGAATCTCTCAGCTCC

Notes: Underlined regions are complementary sequences to HPH-F/R and P3/P4, respectively; wavy lines indicate attB1 and attB2 for Gateway BP reaction. Dashed lines indicate restriction enzyme recognition sites.

We used BLAST tools for similarity analyses and to identify homologs [27]. The ProtParam tool (http://web.expasy.org/cgi-bin/protparam/protparam) was used to determine certain characteristics of the protein encoded by *VdPR1*, including the molecular weight (Mw) and isoelectric point (pI) [28]. We used Y-Loc (http://www.abi.inf.uni-tuebingen.de/Services/YLoc/) to evaluate the subcellular localization of the protein [29], and SignalP 4.1 (http://www.cbs.dtu.dk/services/SignalP/) to predict signal peptides [30]. TMHMM 2.0 (http://www.cbs.dtu.dk/services/TMHMM/) and big-PI Predictor (http://mendel.imp.ac.at/sat/gpi/fungiserver.html) were used to detect transmembrane structures and GPI anchor points, respectively [31, 32].

### Vector construction and fungal transformation

Two novel binary vectors used in this study were constructed on the backbone of the pGKO_2_-gateway vector (deletion vector) [33] and the pSULPH-gfp vector (complementary vector) [34]. Table 1 shows the primers used for vector construction. Generation of a knockout-fusion fragment consisted of three steps: specific amplification, fusion PCR, and nested amplification. A 1.2 kb upstream fragment (UP) and a 1.2 kb downstream fragment (DOWN) were amplified from Vd080 genomic DNA with the primer pairs P1/P3 and P4/P6, respectively. HPH-F and HPH-R primers were designed to amplify the hygromycin cassette (HPH) from the pCTHyg vector. The three amplicons UP, HPH and DOWN at a ratio of 1:3:1, were fused into one fragment by a fusion PCR reaction targeting the reverse complementary adaptor between P3 and HPH-F and between P4 and HPH-R [35]. Subsequently, a nested PCR was performed with the fusion fragment as the template and the primers P2 and P5, which contained Gateway BP reaction adaptors. Then, the final amplified fragment was cloned into the pGKO_2_-gateway vector by a BP recombinant reaction to obtain pGKO_2_-VdPR1 [33].

We conducted ATMT of *V*. *dahliae* Vd080 using AGL-1 harboring pGKO_2_-VdPR1, as described previously [36]. A co-culture of *V*. *dahliae* spores (5 × 10^6^ CFU/mL) and *A*. *tumefaciens* cells (OD = 0.3–0.4) carrying the knockout vector was incubated on nitrocellulose filters (Amersham Pharmacia, Piscataway, NJ, USA) at 25°C for 48 h. Then, the filters were transferred to PDA medium containing 50 μg/mL spectinomycin, 50 μg/mL cefotaxime, 50 μg/mL hygromycin, and 50 μmol/L 5-fluoro-2-deoxyuridine (F_2_dU) (Sigma, St Louis, MO, USA), and incubated until colonies appeared. The nucleoside analog F_2_dU excluded ectopic transformants [33].

For the complementation assay, we carried out restriction enzyme digestion and ligation. A 3.8 kb PCR product containing a 1.0 kb upstream sequence, the full-length *VdPR1* gene coding region, and a 500 bp downstream sequence was amplified from Vd080 genomic DNA using the primers COM-F/R (Table 1). The product was cloned into the *Xma* I/*BsrG* I sites of the binary vector pSULPH-gfp with lac promoter element, thereby conferring chlorimuron-ethy1 resistance and introducing the *GFP* gene [34]. The positive clones were named pSUL-VdPR1. The *VdPR1* complementary vector pSUL-VdPR1 was introduced into the gene deletion mutant ΔVdPR1 via ATMT. Positive transformants resistant to chlorimuron-ethy1 were subjected to single-spore isolation.

### Confirmation of *VdPR1* deletion and complementation mutants

Initial screening of transformants with a deleted *VdPR1* gene or deletion mutants with a re-introduced functional copy of the gene was conducted using a PCR strategy with specific primers (Table 1). Primers HPH-F/R were designed to detect the hygromycin resistance cassette that replaced the *VdPR1* gene in the deletion mutants, and primers Test-F/R were used to amplify a 530 bp region within *VdPR1*. Positive ΔVdPR1 knockout recombinants tested positive for the HPH-targeted fragment, but not for the *VdPR1*. The successfully complemented mutants were identified with COM-F/R and GFP-F/R, and by their chlorimuron-ethy1 resistance. Green fluorescence of positive complemented colonies was visualized under a compound microscope equipped with a GFP filter. Transcript levels of the *VdPR1* gene were determined by quantitative RT-PCR analysis using mutant cDNA as the template and RT-F/R primers. Vdβt-F/R primers were used to detect *V*. *dahliae β-tubulin* as an endogenous control.

### Analysis of vegetative growth, conidiation, and microsclerotia formation of mutants

Mutants derived from Vd080 were characterized to determine their developmental and morphological characteristics. For each sample, 5 μL conidial suspension (1 × 10^7^ CFU/mL) was inoculated on PDA medium and incubated at 25°C. The colony diameters and morphology of vegetative mycelia were examined at 3, 5, 7, 9, and 11 days post-inoculation (dpi). For germination tests, 20 μL spore suspension (1 × 10^4^ CFU/mL) of Vd080 and its mutants were inoculated on solid Czapek-Dox medium and incubated at 25°C. Samples were examined at 4, 8, and 12 hours post-inoculation (hpi) and germination rate was determined by counting 50–60 conidia. To estimate conidia production, 100 μL fresh spore suspension (1 × 10^7^ CFU/mL) of each strain was inoculated into 1900 μL liquid Czapek-Dox medium in a 5 mL tube, and then incubated at 25°C with shaking at 150 rpm. After 7 days, a 10 μL drop of the conidial suspension was placed onto a hemocytometer, and the spores were counted under a microscope.

To determine the influence of cotton root extract on spore production of the mutants, 15 g cotton root was ground in liquid nitrogen, and then extracted in 70 mL deionized water with shaking for 1 h. Then, the mixture was filtered through a 0.22-μm microfiltration membrane before use. For each strain, 100 μL fresh spore suspension (1 × 10^7^ CFU/mL) was inoculated into 1900 μL cotton root extract solution in a 5 mL tube. The mixture was incubated at 25°C with shaking at 150 rpm. The spores were counted at 24, 48, and 96 hours post-inoculation (hpi) [37].

The carbon source utilization of mutants was determined using four different monosaccharides or polysaccharides. Sucrose (30 g/L), skim milk powder (18 g/L), cellulose (5 g/L), and starch (1 g/L) were individually added to Czapek-Dox medium lacking sucrose. Then, 5 μL conidial suspension (1 × 10^7^ CFU/mL) of each strain was placed in the center of a plate of each different medium, and then incubated at 25°C in the dark. The colony diameters and morphology were determined at one day intervals. All of the tests were repeated three times.

### Pathogenicity assays

Cotton (cv. Jimian 11) plants were used in infection assays to evaluate the effect of the *V*. *dahliae* wild-type strain Vd080 and its mutants on virulence. This cultivar is highly susceptible to Verticillium wilt. The inoculum concentration was adjusted to 1 × 10^7^ CFU/mL. Each strain was inoculated onto 8–10 cotton seedlings at the first euphylla stage by immersing the seedling roots in the conidial suspension. This experiment was replicated three times [38]. The inoculated cotton seedlings were cultivated in a standard greenhouse at 25–30°C under a 16-h/8-h light–dark photoperiod. Disease progress was recorded from 7 to 24 dpi. The disease index (DI) was calculated based on a five-scale categorization of Verticillium wilt disease on cotton seedlings [38].

### qPCR quantification of fungal biomass in plant tissue

To investigate the colonization ability of the wild-type strain Vd080 and its mutants, fungal biomass in infected cotton plants was estimated using qPCR of fungi-plants mixed DNA which was extracted from roots and hypocotyl. At 1, 2, 3, 4, 7, 14 and 21 dpi root and hypocotyl were harvested by cutting with a sharp scissors, plant tissues were sterilized for 10 min with 70% ethanol and rinsed three times with sterile water, three cotton plants as a treatment. Tissue samples were ground to a fine powder in the presence of liquid nitrogen and total DNA was extracted with Dellaporta protocol [39]. DNA concentration of each sample was measured by Nano Drop 2000 (Thermo scientific corporation) and adjusted to 100 ng/μL for qPCR reaction. Primer Vdβt-F and Vdβt-R were designed based on *V*. *dahliae β-tubulin* gene (Table 1). The endogenous control *Gossypium hirsutum Actin* gene was amplified using Primer Act-F and Act-R (Table 1). A qPCR reaction was performed using the QuantiFast SYBR Green PCR (Qiagen, Valencia, CA, U.S.A.) master mix, PCR cycling started with an initial step of 95°C denaturation for 10 min, 40 cycles at 95°C for 30 s, 60°C for 30 s, and 72°C for 30 s to calculate cycle threshold values, followed by 95°C for 15 s, 60°C for 1 min, 95°C for 15 s and 60°C for 15 s to obtain a melting curve. The data were analyzed using the 2^–ΔΔCt^ relative quantification method [40]. The primer specificity and the formation of primer-dimers were monitored by dissociation curve analysis.

### Gene expression analysis

To monitor the transcriptional expression profile of *VdPR1* in the wild-type strain Vd080 during cotton infection, the roots were harvested at 1, 2, 3, 4, and 7 dpi and flash frozen in liquid nitrogen for RNA extraction. *V*. *dahliae* cultured on PDA medium without being used to inoculate cotton plants was set as the control. After grinding, 100 mg of ground material was used for total RNA extraction and cDNA synthesis. The transcript level of *VdPR1* was determined using RT-qPCR, as described above.

## Results

### *VdPR1* cloning and bioinformatics analysis

**Fig 1:**
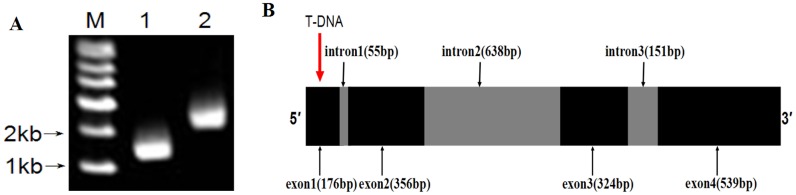
Cloning and gene structure of *VdPR1*. **A)** Electrophoretogram of *VdPR1* cloning process. M: marker; lane 1: ORF of *VdPR1* cloned from cDNA of Vd080; lane 2: *VdPR1* cloned from genomic DNA of Vd080. **B)** Schematic diagram showing position of T-DNA insert in vdpr1 mutant and structure of *VdPR1* gene.

In a previous study, the vdpr1 mutant was confirmed by Southern hybridization to have a single copy of the T-DNA insert [21]. Based on the T-DNA flanking regions and comparisons with sequences in the VdLs.17 reference genome database, the functional gene disrupted by T-DNA in vdpr1 was found to be highly homologous to VDAG_00904 (100% homology). The T-DNA insert was located in the first exon of VDAG_00904 (*VdPR1*). Further analysis of gene structure revealed that the full-length *VdPR1* gene consisted of 2239 bp, with four exons and three introns (Fig 1).

The function of the deduced *VdPR1* protein was identified by similarity analysis and by comparison with its homologs with the BLAST program. The VdPR1 protein showed 100% identity with VDAG_00904, but no more than 80% identity with any other annotated gene in the NCBI non-redundant protein sequence database. The hypothetical protein contained a conserved C-terminal PA14 superfamily domain, which is related to yeast adhesins and some bacterial toxins [41]. Its molecular mass and pI were 49 kD and 9.23, respectively. The Y-Loc analysis predicted that the subcellular location of VdPR1 was in the secretome pathway. The SignalP 4.1 analysis identified the N-terminal signal sequence as a 19 a.a. peptide fragment. No transmembrane regions or GPI anchor points were identified by TMHMM 2.0 and big-PI Predictor, respectively. These results suggest that *VdPR1* may be specific to *V*. *dahliae*, and that it encodes a secreted protein.

### Generation of *VdPR1* knockout and complemented mutants

**Fig 2:**
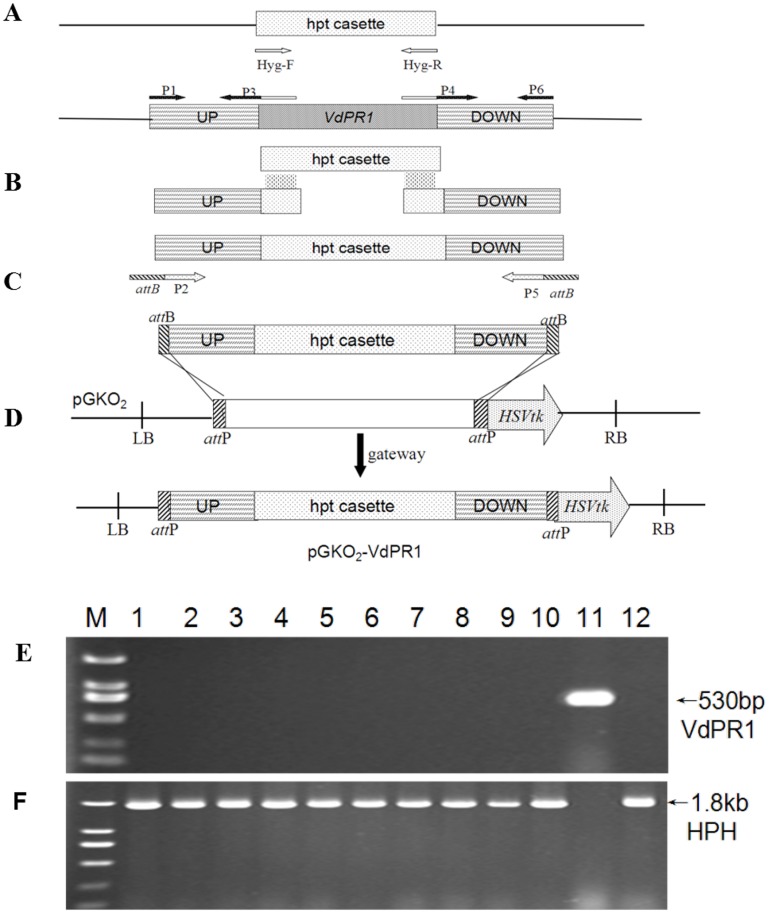
Strategy for construction of gene deletion vector and molecular identification of positively deleted ΔVdPR1 mutants. **A)** Amplification of HPH cassette, upstream fragment (UP) and downstream fragment (DOWN). **B)** UP, HPH, DOWN fused into one fragment. **C)** Nested PCR amplification. **D)** BP recombinant reaction. **E)** Amplification of *VdPR1* gene. Lanes 1–10: ΔVdPR1 mutant; lane 11: Vd080; lane 12: knockout vector. **F)** Amplification of HPH gene. Lanes 1–10: ΔVdPR1 mutant; lane 11: Vd080; lane 12: knockout vector.

To investigate the function of *VdPR1* in *V*. *dahliae*, we first generated *VdPR1* deletion mutants by replacing the ORF of *VdPR1* with a hygromycin resistance cassette in a gene construct, and then transforming this construct into the wild-type *V*. *dahliae* strain Vd080 using the ATMT method [42] (Fig 2A, 2B, 2C and 2D). *HSVtk*, which was located on the pGKO_2_-VdPR1 vector, acted as a negative selection marker against ectopic transformants. The *VdPR1* gene was deleted in 10 out of 20 transformants analyzed by PCR, giving a 50% positive transformant efficiency rate. An HPH fragment, but not the *VdPR1* sequence, was amplified from ΔVdPR1 mutants (Fig 2E and 2F).

**Fig 3:**
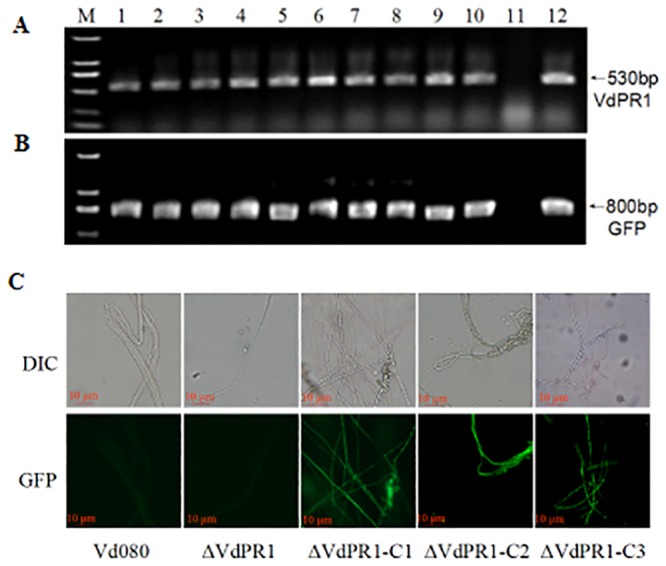
Screening of complementary mutants ΔVdPR1-C. **A)** Amplicons of *VdPR1*. Lanes 1–10: complementary mutant; lane 11: ΔVdPR1; lane 12: complementary vector. **B)** Detection of GFP. Lanes 1–10: complementary mutant; lane 11: ΔVdPR1; lane 12: complementary vector. **C)** Visualization of green fluorescence under visible-light and fluorescence microscopes, the complemented mutants which were tagged with a green fluorescent protein (GFP) gene. Bar = 10 μm.

To confirm that the phenotypic differences observed in gene-deletion mutants were associated with the gene replacement event, a complete functional copy of *VdPR1* including the native promoter and terminator elements was integrated into the binary vector pSULPH-gfp, and then transformed into the ΔVdPR1 mutants. This resulted in the complemented strain ΔVdPR1-C. We obtained 20 chlorimuron-ethy1 resistant transformants, and randomly selected 10 for molecular identification. PCR analyses confirmed that *VdPR1* was absent from the ΔVdPR1 mutant but present in the gene-complemented strains (Fig 3A and 3B). Under a fluorescence microscope, the mycelia of all ΔVdPR1-C isolates displayed significant green fluorescence that differed from that observed in the mycelia of Vd080 and the ΔVdPR1 mutant (Fig 3C).

**Fig 4:**
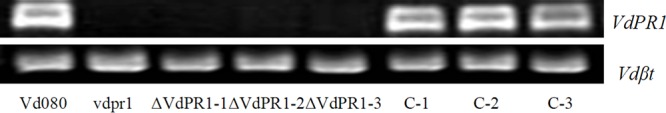
RT-PCR analysis of *VdPR1* transcripts in deletion and complementation mutants. Note: The *V*. *dahliae β-tubulin* (*Vdβt*) was used as a control.

Three deletion mutants (ΔVdPR1-1, ΔVdPR1-2, and ΔVdPR1-3) and three complemented mutants (ΔVdPR1-C1, ΔVdPR1-C2, and ΔVdPR1-C3) were randomly selected for further functional analyses of *VdPR1*. The absence of the *VdPR1* from the deletion mutants was further confirmed by RT-PCR using RT-F/R primers, with the *β-tubulin* gene (*Vdβt*) used as an internal control. As expected, *VdPR1* was not detected in the ΔVdPR1 deletion mutants, but was detected in the ΔVdPR1-C complemented strains at similar transcript levels as that in the wild-type Vd080 (Fig 4).

### *VdPR1* is associated with microsclerotia development, mycelial growth, and spore production

**Fig 5:**
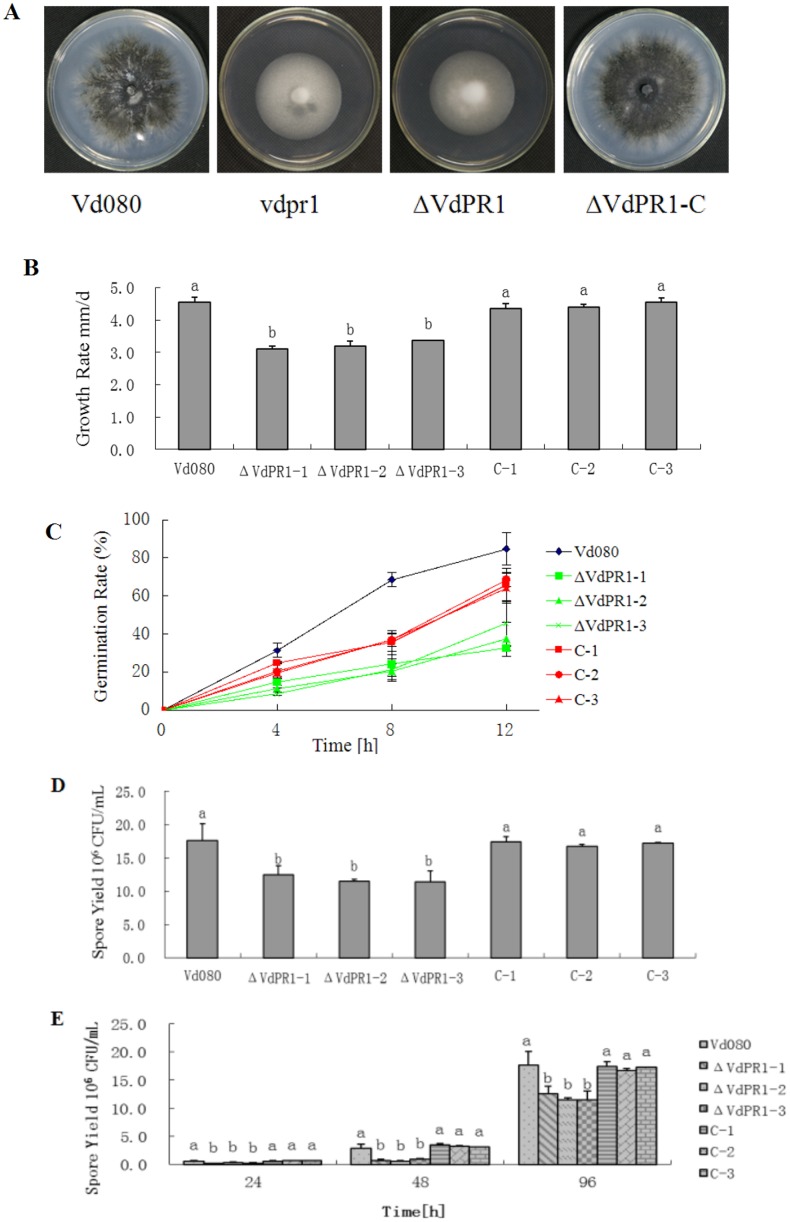
Phenotypic characterization of wild-type Vd080 strain and mutants. **A)** Colony morphology of wild-type strain and mutants on potato dextrose agar at 11 days post inoculation. **B)** Radial growth rates of isolates. **C)** Spore germination of mutants on solid Czapek-Dox medium. **D)** Spore yield of mutants in liquid Czapek-Dox medium. **E)** Effect of cotton root extract solution on spore production of mutants. Means and standard errors were calculated from at least three independent experiments, with a significant difference of *P* < 0.05 among strains.

To analyze the function of *VdPR1* in fungal growth and microsclerotia production, the wild-type strain Vd080 and its mutants were grown on PDA medium. The colony morphology of the wild-type and complemented strains were very similar with abundant microsclerotia and a high concentration of melanin. In contrast, the knockout mutant was light-colored and did not produce microsclerotia (Fig 5A). The growth rate of the deletion mutant ΔVdPR1, as measured by the increase in colony diameter per day, was 82% of that of the wild-type Vd080 (4.55 mm/d). The growth rates of the ΔVdPR1-C complementary mutants were rescued to that of the wild-type Vd080 (Fig 5B). Conidia of mutant ΔVdPR1 germinated more slowly than those of Vd080 and ΔVdPR1-C (Fig 5C). After 8 h of incubation in liquid Czapek-Dox medium about 22% of ΔVdPR1 conidia were germinated compared to 69% of Vd080 conidia and 37% of ΔVdPR1-C conidia. Then, at 12 hpi, the germination rate was 38% for ΔVdPR1 with 85% for Vd080 and 66% for ΔVdPR1-C. In addition, the spore concentration was also significantly lower in ΔVdPR1 (11.45 × 10^6^) than in the wild-type Vd080 (17.65 × 10^6^) with decreasing 35%, and the spore concentration of ΔVdPR1-C (17.43 × 10^6^) has no significant difference with the wild type Vd080 (Fig 5D).

To further explore the interaction between this pathogen and its host, we investigated whether *VdPR1* plays a role in spore production of *V*. *dahliae* induced by cotton root extract. Interestingly, spore production began to increase sharply at 24 hpi in cotton root extract, in both the wild-type and mutant strains. Compared with the initial spore production rate of each respective line, wild-type Vd080 produced 60 times more spores and knockout mutants increased 23–40 times more spores on cotton root extract (Fig 5E), compared with 5–6 times more spores in liquid Czapek-Dox medium. From 24 hpi to 48 hpi, the spore production of ΔVdPR1 mutants was significantly lower than that of the wild-type strain Vd080 with reducing 53%-75%.

### Protease and cellulase activities of *VdPR1*

**Fig 6:**
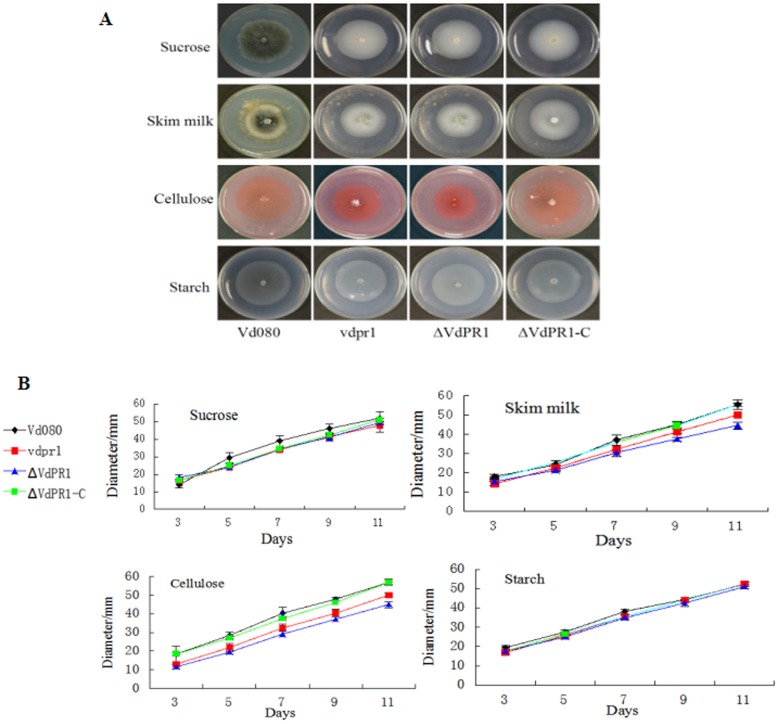
Culture characteristics and growth rates of isolates on media with different carbon sources. **A)** Phenotypes of mutants and Vd080. **B)** Radial growth rates of isolates. Means and standard errors were calculated from at least three independent experiments, with a significant difference of *P* < 0.05 among strains.

To investigate the role of *VdPR1* in carbon utilization, we compared the radial growth rates of the wild-type strain Vd080 and its mutants on media containing sucrose, skim milk, cellulose, or starch. Growth of the mutants was not affected on media containing sucrose and starch, suggesting that *VdPR1* was not involved in sucrase or amylase activities. All of the ΔVdPR1 knockout mutants showed impaired growth on media containing skim milk and cellulose; their growth was reduced by 15% and 21%, respectively, compared with the growth of the wild-type Vd080 on these media. The complemented strains showed protease and cellulase activities almost comparable to those of the wild-type Vd080 (Fig 6).

### *VdPR1* is required for virulence

**Fig 7:**
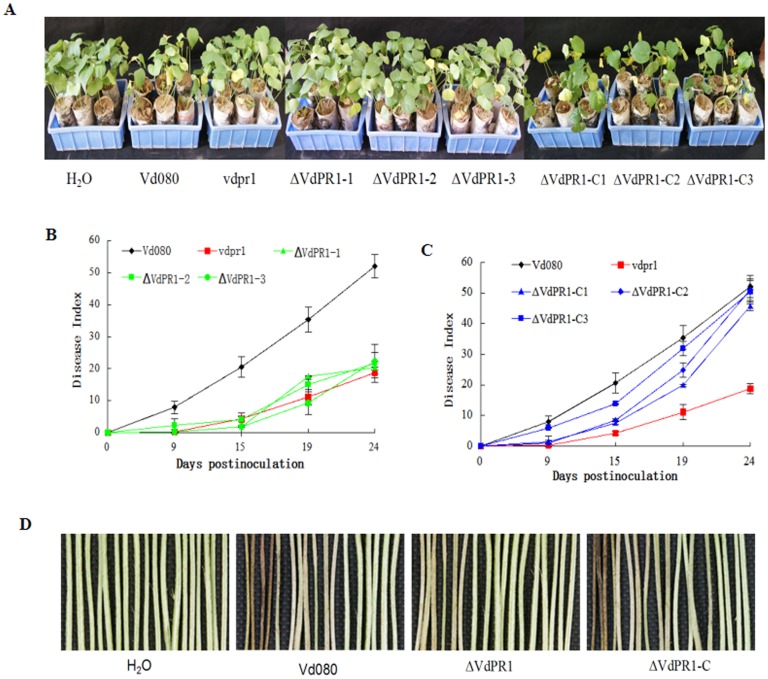
Pathogenicity analysis of mutants and Vd080. **A)** Cotton plants at 24 days post inoculation with Vd080 and its mutants. **B)** Progress of disease symptoms caused by *VdPR1* knockout mutants and Vd080. **C)** Progress of disease symptoms caused by *VdPR1* complemented mutants and Vd080. **D)** Vascular discoloration of cotton plants inoculated with Vd080 and its mutants.

Next, we evaluated whether *VdPR1* contributes to pathogenicity. Cotton seedlings were inoculated with a spore suspension of the wild-type Vd080, its deletion mutants, its complemented mutants, and the T-DNA insertion mutant vdpr1 as control. Disease symptoms appeared earlier in plants inoculated with the wild-type Vd080 strain than in those inoculated with ΔVdPR1. The cotton plants inoculated with wild-type Vd080 showed severe chlorosis and stunting in some leaves at 7 dpi, while those infected with the ΔVdPR1 knockout mutant showed slight chlorosis in a few leaves at 9 dpi. At 24 dpi, disease symptoms were apparent on plants inoculated with Vd080, but were much less severe on plants inoculated with ΔVdPR1. The DI of ΔVdPR1 (21.48 ± 1.0) was significantly lower than that of the wild-type Vd080 (52.11 ± 3.7) reducing by 59% (Fig 7A and 7B), The plants inoculated with ΔVdPR1-C showed disease symptoms as severe as those caused by the wild-type Vd080. The DI values of the three complemented mutants ranged from 45.64 ± 1.4 to 51.03 ± 3.5, implying that the virulence of the complemented strain ΔVdPR1-C had been restored to the wild-type level (Fig 7A and 7C). These results indicated that *VdPR1* is important for the pathogenicity of *V*. *dahliae*.

*V*. *dahliae* causes vascular wilt in host plants, and browning of vascular tissue. To investigate further the reduced pathogenicity of the *VdPR1* deletion mutant, we observed the stem vascular bundles of plants inoculated with wild-type Vd080 and its mutants. As expected, most cotton plants inoculated with Vd080 or complemented ΔVdPR1-C exhibited vascular wilt symptoms and brown stems, whereas only a few vascular bundles showed browning symptoms in plants infected with ΔVdPR1 (Fig 7D).

**Fig 8:**
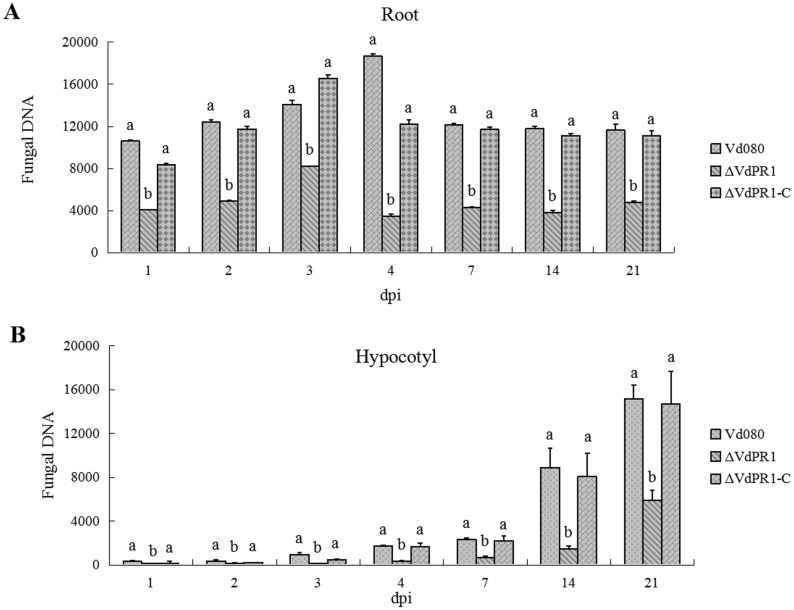
Detection of fungal biomass in infected cotton tissues. **A)** Quantification of mutant DNA in root section. **B)** Quantification of mutant DNA in hypocotyl section. The *V*. *dahliae β-tubulin* gene was used as a detect gene, the *Gossypium hirsutum Actin* gene was used as an endogenous control gene. Error bars indicate standard error (*n* = 3).

To evaluate the roles of *VdPR1* in initial colonization ability and systemic infection, we quantified the fungal DNA of the wild-type Vd080 and its mutants in cotton plants by qPCR. In the roots of infected cotton plants, the biomass of the pathogen increased during early days, then downgraded to steady. The fungal biomass of the wild-type Vd080 reached its maximum quantity at 4 dpi, while the biomass of ΔVdPR1 and ΔVdPR1-C peaked at 3 dpi. In the hypocotyl tissue of the infected plants, the biomass of both the wild-type Vd080 and its mutants increased over time. However, the biomass of the ΔVdPR1 knockout mutant was always significantly lower than those of the wild-type Vd080 and the ΔVdPR1-C strain in root and hypocotyl tissues, while biomasses of the wild-type Vd080 and ΔVdPR1-C were not significantly different. These results demonstrated that the reduced initial colonization ability of ΔVdPR1 is one of essential factors related to impaired pathogenicity (Fig 8A and 8B).

**Fig 9:**
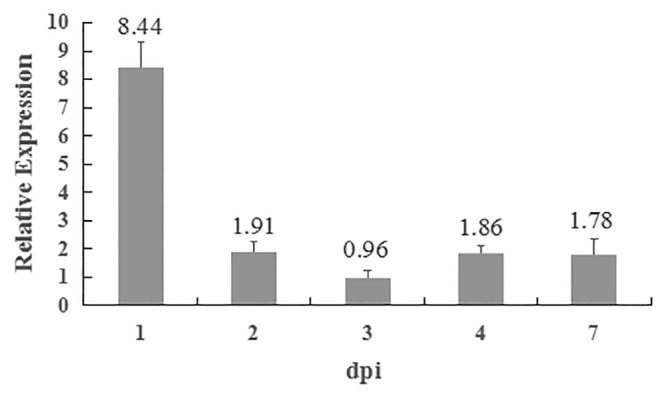
Transcriptional expression analysis of *VdPR1* during the *V*. *dahliae* response to cotton. Hyphae collected from PDA medium and the expression of *VdPR1* was set to 1. The *V*. *dahliae β-tubulin* was used in relative expression analyses. Error bars indicate standard error (*n* = 3).

To determine whether *VdPR1* is related to *V*. *dahliae* pathogenicity, the expression level of *VdPR1* was detected during the cotton infection process by RT-qPCR. As expected, the expression of *VdPR1* was continuously up-regulated after infection of cotton, the transcript level was significantly elevated by over 8-fold at 1 dpi (Fig 9). These results suggest that *VdPR1* may be involved in *V*. *dahliae* pathogenicity and that it plays a curial role in the early infection process.

## Discussion

In this study, the function of *V*. *dahliae* gene *VdPR1* was investigated and analyzed in both biological characteristics and pathogenicity by knock out and complementary strategy. Deletion of *VdPR1* reduced fungal growth, conidiospore production and microsclerotia production, and decreased the capacity for initial infection and systemic spread through the xylem. The lower conidia germination rate of ΔVdPR1 and decreased conidia production in media containing the cotton root extract could explain the above observed symptoms (Fig 5C and 5E). Our data showed that *VdPR1* plays a key role in the development and virulence of *V*. *dahliae*. The BLASTP analysis showed that there were no homologous genes with more than 80% identity in the non-redundant protein sequence database, except for VDAG_00904 (VdLs.17), which showed 100% homology. These results support that *VdPR1* may be a new gene of *V*. *dahliae* with unknown function.

Deletion of *VdPR1* from *V*. *dahliae* resulted in mutants with severely impaired virulence to cotton, with a 2-day delay in infection and an 88% reduction of symptoms (Fig 7A and 7B), compared with those caused by wild-type Vd080. The fungus *Magnaporthe grisea* attaches to plant roots and enters the plants directly by forming an appressorium invasion structure, whereas the attachment of *Verticillium* species depends on particular molecules, such as adhesins [15, 43, 44]. Bioinformatics analyses of *VdPR1* showed that the protein contains a PA14 superfamily domain at the C-terminal. This domain is present in bacterial beta-glucosidases and in other glycosidases, glycosyltransferases, proteases, amidases, yeast adhesins, and some bacterial toxins [41]. The absence of the VdPR1 protein resulted in impaired pathogenicity in ΔVdPR1. We also investigated the role of *VdPR1* in the invasion and growth of *V*. *dahliae*. We quantified the fungal biomass of the wild-type Vd080 and its mutants in the root and hypocotyl of cotton plants. These analyses showed that the ΔVdPR1 mutant was deficient in its ability to colonize the root, which resulted in impaired spread systemically through the xylem (Fig 8A and 8B), partly similar to the deletion mutants of *VdSNF1*, *VGB* and *Vta2* that were unable to systemically colonize the host plant [9, 13, 15]. Furthermore, *VdPR1* was also observably continuously up-regulated during cotton infection (Fig 9). Together these findings suggest that *VdPR1* is important for root initial infection and indirect contribution to subsequent colonization of vascular tissues. Which confirm that *VdPR1* is involved in *V*. *dahliae* pathogenicity.

The durable resistant structures of the fungus, microsclerotia are considered to be an important model for exploring the initial penetration and pathogenicity mechanisms in *V*. *dahliae* [45]. A comparison of microsclerotia development and pathogenicity-related genes showed that microsclerotia development is not absolutely related to pathogenicity [12, 46], but it is the first step in host invasion and initiation of years of epidemic wilt diseases in the fields. Previous studies have shown that certain pathogenic factors affect microsclerotia production and melanin accumulation, thereby determining the virulence of the pathogen [7]. Consistent with those findings, the ΔVdPR1 mutant that lacked the *VdPR1* gene produced no microsclerotia and accumulated less melanin than did wild-type Vd080 (Figs 5A and 6A). These findings are consistent with the role of *VdPR1* in the network controlling virulence and microsclerotia formation.

Generally, CWDEs play an important role in pathogenicity of *V*. *dahliae* by degrading the plant cell wall. During the initial interaction between fungal pathogens and their plant hosts, a key step for host penetration and systemic infection is to overcome the natural barrier of the cell wall. *F*. *oxysporum* and *C*. *carbonum* produce a variety of carbohydrate-active enzymes (CAZymes) including pectinases, cellulases, and proteases, which degrade plant polysaccharide materials [47, 48]. Pectinases and cellulases were shown to be critical for the induction of symptoms and pathogenesis of *V*. *dahliae* [1]. Proteases allow the pathogen to penetrate the plant cell wall during the colonization process-by degrading host defense-related proteins, and allowing the pathogen to use plant proteins as a nutrient source [49]. In this study, *VdPR1* was shown to be associated with plant cell wall degradation. The radial growth rates of the *VdPR1* knockout mutant ΔVdPR1 on cellulose or skim milk were reduced by 21% and 15%, respectively (Fig 6A and 6B), compared with those of the wild-type Vd080. In *Sclerotinia sclerotiorum*, many secreted proteins belonging to the CWDE family act as pathogenicity or virulence factors [50]. Similar to the *V*. *dahliae* secreted protein VdSSP1, the deduced VdPR1 protein has a signal peptide that targets it to the secretome pathway. This result supports the hypothesis *VdPR1* is a secreted virulence factor belonged to the CWDE family, however, the specific location of VdPR1 has not been verified experimentally. To figure out the role of VdPR1 in related signaling pathways and the interaction between the pathogen and the host plant will be conducted in future studies.
